# One-pot three-component synthesis of quinoxaline and phenazine ring systems using Fischer carbene complexes

**DOI:** 10.3762/bjoc.6.52

**Published:** 2010-05-25

**Authors:** Priyabrata Roy, Binay Krishna Ghorai

**Affiliations:** 1 Department of Chemistry, Bengal Engineering and Science University, Shibpur, Howrah 711103, India

**Keywords:** azaisobenzofuran, Diels–Alder, Fischer carbene complex, phenazine, quinoxaline

## Abstract

One-pot three-component coupling of *o*-alkynylheteroaryl carbonyl derivatives with Fischer carbene complexes and dienophiles leading to the synthesis of quinoxaline and phenazine ring systems has been investigated. This involves the generation of furo[3,4-*b*]pyrazine and furo[3,4-*b*]quinoxaline as transient intermediates, which were trapped with Diels–Alder dienophiles. This is the first report on furo[3,4-*b*]pyrazine intermediates.

## Introduction

**Scheme 1:**
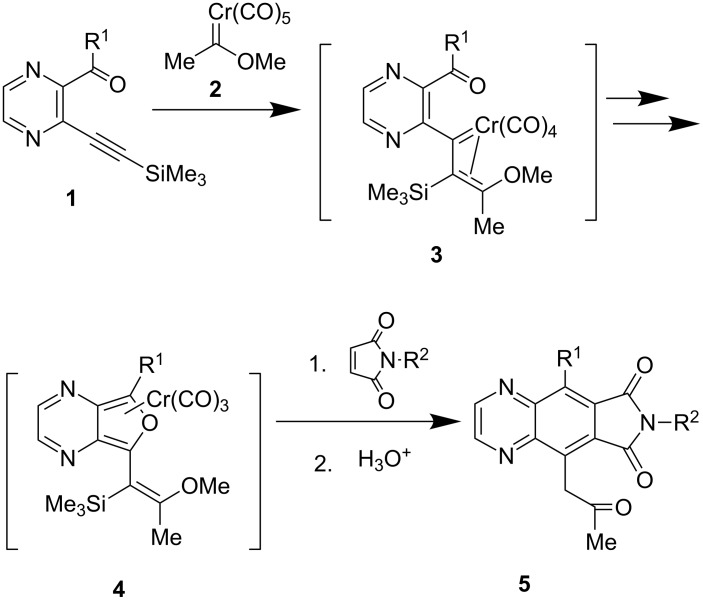
Synthetic plan towards quinoxaline derivatives.

Nitrogen-containing heterocycles are abundant in nature and exhibit diverse and important biological properties [1]. Quinoxaline and phenazine derivatives are important classes of nitrogen containing heterocycles which exhibit a wide range of biological activities. Many phenazine compounds are found in nature and are produced by bacteria such as *Pseudomonas* spp., *Streptomyces* spp. and *Pantoea agglomerans*. These phenazine natural products have been implicated in the virulence and competitive fitness of the parent organisms [2–3]. These compounds show diverse biological activities such as antibacterial, antifungal, antiviral and antitumor properties [4–8]. While rarely found in nature, quinoxalines are well known in the pharmaceutical industry and have been shown to possess a broad spectrum of biological activity including antiviral and antibacterial properties and also act as kinase inhibitors [9–11]. These heterocyclic ring systems are most commonly assembled by the annulation of a heterocyclic ring onto a pre-existing benzene ring [12–21]. A less common approach to these ring systems is the annulation of benzene rings onto pre-existing heterocyclic rings [22]. This manuscript focuses on the successful execution of the latter transformation through a multicomponent reaction process to access these ring systems (Scheme 1). The synthetic approach involves a simultaneous one-pot construction of quinoxaline or phenazine rings which occurs in conjunction with the tandem generation and trapping of an azaisobenzofuran intermediate [23–26]. The synthesis of quinoxaline ring systems involves the coupling of Fischer carbene complexes [27–32] with 2-alkynyl-3-pyrazine carbonyl derivatives, followed by the generation of a hitherto unknown intermediate e.g. furo[3,4-*b*]pyrazine **4** and trapping of the latter with dienophiles. Phenazine derivatives can be synthesized using similar methodology from the coupling of 2-alkynyl-3-quinoxaline carbonyl derivative through the generation and trapping of furo[3,4-*b*]quinoxaline intermediates [22].

## Results and Discussion

**Scheme 2:**
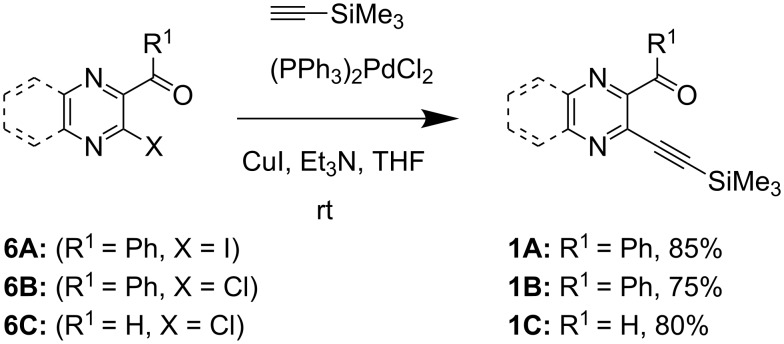
Preparation of *o-*alkynyl carbonyl derivatives **1**. **A:** pyrazine series; **B,C:** quinoxaline series.

Our investigation commenced with the synthesis of *o-*alkynyl carbonyl derivatives **1**, which were prepared in good yield from the iodoketone **6A** or chloroketone **6B** [33] or chloroaldehyde **6C** [34] using palladium catalyzed Sonogashira coupling reactions as depicted in Scheme 2. Iodoketone **6A** was prepared in 80% yield from (3-chloro-2-pyrazinyl)phenylmethanone [35] by halogen exchange with NaI in acetonitrile.

The three component coupling reaction of pyrazinyl ketone **1A,** carbene complex **2** and *N*-phenylmaleimide (~ 1:1:1 ratio) in refluxing THF was initially investigated (Table 1, entry 1). This reaction led to a mixture of oxanorbornene derivative **7a** and quinoxaline derivative **5a** through the tandem generation and trapping of the furo[3,4-*b*]pyrazine intermediate **4** (R^1^ = Ph). Ring opening followed by extrusion of water by treatment of **7a** with DBU in refluxing toluene, gave the quinoxaline derivative **5a** [36]. The stereochemistry of the adduct **7a** was assigned as *exo* based on the chemical shift of H_A_ and H_B_ (<4 ppm) [27]. A similar reaction process using *N*-methylmaleimide (entry 2) as dienophile led to the quinoxaline derivative **5b** as the sole product after exposure to mild acid.

**Table 1:** Synthesis of quinoxaline and phenazine derivatives.

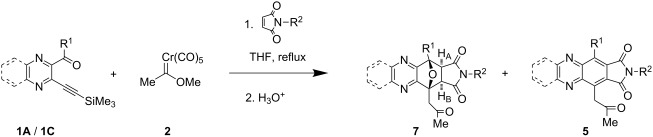					

Entry	Carbonyl compounds	R^1^	R^2^	Products (yield^a^)	

1	**1A**	Ph	Ph	**7a** (42%)	**5a** (30%)
2	**1A**	Ph	Me	-	**5b** (55%)
3	**1C**	H	Ph	**7c** (10%)^b^	**5c** (52%)
4	**1C**	H	Me	-	**5d** (55%)

^a^Isolated yield. ^b^Contaminated with **5c**.

The three component coupling reaction of *o*-alkynyl quinoxaline carbonyl derivative **1C**, carbene complex **2** and *N*-phenylmaleimide/*N*-methylmaleimide was also examined (Table 1, entry 3 & 4). In these cases, tandem generation and trapping of the desired furo[3,4-*b*]quinoxaline intermediates proceeded smoothly to give the corresponding hetero-polyaromatic phenazine derivatives **5c/5d**. Although the [4 + 2] oxa-bridged adduct **7c** was isolated, but it was contaminated with **5c** since **7c** readily converts to **5c** in chloroform at room temperature (Table 1, entry 3).

**Scheme 3:**
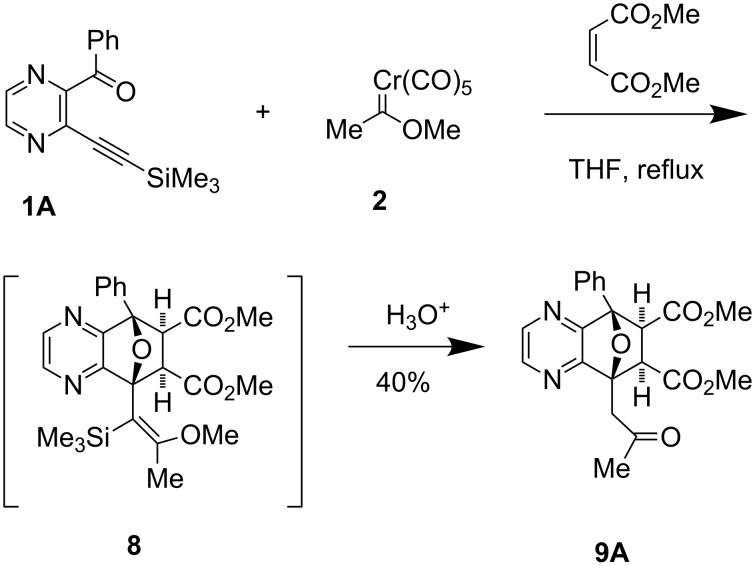
Synthesis of quinoxaline derivative.

The reaction was also examined with dimethyl maleate as the dienophile (Scheme 3). The reaction of pyrazinyl ketone **1A**, carbene complex **2** and dimethyl maleate under the same conditions as previously described afforded the three component coupling product **9A** in 40% yield via the unstable enol ether **8**. No aromatized product was isolated, even under mild acidic conditions.

**Scheme 4:**
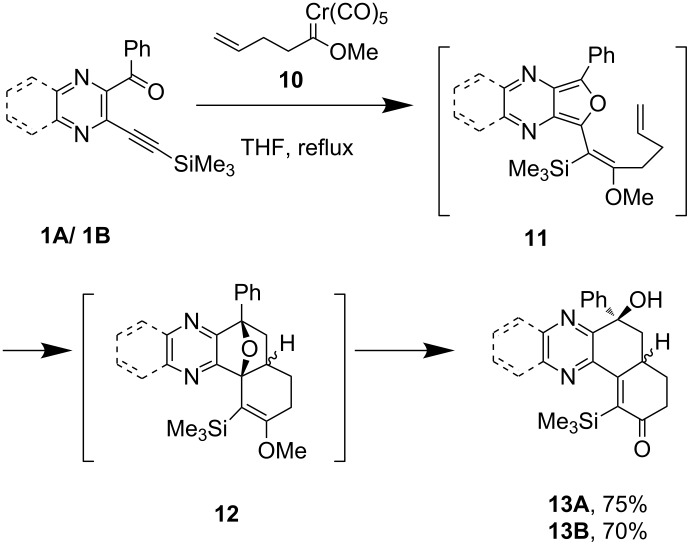
Synthesis of azahydrophenanthrone derivatives.

As part of a general effort to prepare aza-analogues of hydrophenanthrene natural products (including morphine alkaloids and abietanes) and tetracyclic triterpenes, the coupling of *o-*alkynyl pyrazine/quinoxaline carbonyl derivatives **1A**/**1B** with simple γ,δ-unsaturated Fischer carbene complex **10** was investigated. This reaction proceeds via a tandem process involving the formation of azaisobenzofuran **11**, followed by intramolecular Diels–Alder reaction, and ring opening of **12** to afford azahydrophenanthrone derivatives **13A**/**13B** exclusively, in satisfactory yield (Scheme 4).

## Conclusion

We have demonstrated a new route for the tandem generation of furo[3,4-*b*]pyrazine/ furo[3,4-*b*]quinoxaline intermediates by the coupling of *o*-alkynylheteroaryl carbonyl derivatives with Fischer carbene complexes. The intermediates can be trapped through Diels–Alder reaction with dienophiles leading to the synthesis of nitrogen containing heterocyclic analogues of quinoxaline and phenazine, respectively, in one-pot. This is the first report of in situ generation of furo[3,4-*b*]pyrazine intermediates.

## Supporting Information

File 1 General procedure for the preparation of *o-*alkynyl carbonyl derivatives **1** and quinoxaline and phenazine derivatives and spectral data for selected compounds.
