# Supplementary material for: One-pot three-component synthesis of quinoxaline and phenazine ring systems using Fischer carbene complexes
Source: Beilstein J Org Chem. 2010 May 25;6:52. doi: 10.3762/bjoc.6.52 (PMC2900911; doi:10.3762/bjoc.6.52)
Supplement: File 1 — General procedure for the preparation of o-alkynyl carbonyl derivatives 1 and quinoxaline and phenazine derivatives and spectral data for selected compounds. [file Beilstein_J_Org_Chem-06-52-s001.pdf]

Supporting Information

for

One-pot three-component synthesis of quinoxaline and phenazine ring systems using Fischer carbene complexes

Priyabrata Roy and Binay Krishna Ghorai*

Address: Department of Chemistry, Bengal Engineering and Science University, Shibpur, Howrah 711103, India

Email: Binay Krishna Ghorai - bkghorai@yahoo.co.in

* Corresponding author

General procedure for the preparation of *o*-alkynyl carbonyl derivatives 1 and quinoxaline and phenazine derivatives and spectral data for selected compounds.

General details:

All melting points are uncorrected. IR spectra were recorded on a Jasco FT/IR-460 plus instrument (KBr or neat). The ^{1}H NMR and ^{13}C NMR spectra were recorded at Bruker-AV500 (500 MHz). Splitting patterns of ^{1}H NMR spectra are designated as s, singlet; d, doublet; t, triplet; q, quartet; m, multiplet; br, broad. LCMS spectra were recorded on a Agilent 6120 mass spectrometer. Unless otherwise noted, all reactions were carried out under an inert atmosphere in flame dried flasks. Solvents and reagents were dried and purified by distillation before use as follows: tetrahydrofuran, toluene, hexane, diethyl ether from sodium benzophenone ketyl; dichloromethane and chloroform from P_2O_5; DMF and diisopropylamine from CaH_2; triethylamine and pyridine from solid KOH. After drying, organic extracts were evaporated under reduced pressure and the residue was column chromatographed on silica gel (Spectrochem, particle size 100-200 mesh), using an ethyl acetate-petroleum ether (60–80 °C) mixture as eluant unless specified otherwise.

1. General procedure I – synthesis of *o*-alkynyl carbonyl derivatives 1

A mixture of carbonyl derivative (1 mmol), $Pd(PPh_3)_2Cl_2$ (0.05 mmol), PPh_3 (0.025 mmol), trimethylsilylacetylene (1.5 mmol) and triethylamine (1.5 mmol) in THF (5 mL) was stirred for 20 min at room temperature, and then CuI (0.01 mmol) added. The reaction was stirred for 12–16 h at room temperature, and the solvent removed on a rotary evaporator. The residue was treated with dichloromethane and filtered through celite. The filtrate was concentrated and the residue purified by chromatography (silica gel/ ethyl acetate: petroleum ether, 1:9).

1.1. Phenyl-(3-trimethylsilanylethynyl-2-pyrazinyl)methanone (1A):

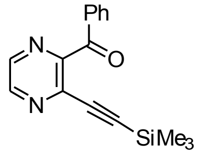

General procedure I was followed using (3-iodo-2-pyrazinyl)phenylmethanone (**6A**) (0.70 g, 2.26 mmol), $Pd(PPh_3)_2Cl_2$ (0.08 g, 0.11 mmol), PPh_3 (0.02 g, 0.06 mmol), trimethylsilylacetylene (0.33 g, 3.38 mmol) and triethylamine (0.34 g, 3.38 mmol) in THF (20 mL), followed by CuI (5 mg, 0.03 mmol). After column chromatography, a brown oil (0.537 g, 85%) identified as compound **1A** was obtained. IR (KBr, cm^{-1}): 2173, 1677; ^{1}H NMR (500 MHz, $CDCl_3$): δ 8.67 (d, 1H, J=2.5 Hz), 8.57 (d, 1H, J=2.5 Hz), 7.88-7.81 (m, 2H), 7.62 (m, 1H), 7.54-7.44 (m, 2H), 0.06 (s, 9H); ^{13}C NMR (125 MHz, $CDCl_3$): δ 192.2, 154.3, 145.0, 141.9, 137.4, 135.3, 134.0, 130.2 (2C), 128.6 (2C), 105.0, 99.3, -0.8 (3C); MS: m/e (relative intensity): 281 (MH^+, 100), 233 (30), 209 (26); Anal. Calcd for $C_{16}H_{16}N_2OSi$: C, 68.54; H, 5.75; N, 9.99. Found: C, 68.48; H, 5.84; N, 9.91.

1.2. 3-Trimethylsilanylethynylquinoxaline-2-carboxaldehyde (1C):

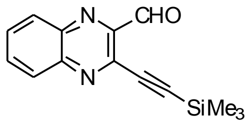

General procedure I was followed using 3-chloroquinoxaline-2-carboxaldehyde(**6C**) [1] (0.37 g, 1.90 mmol), $Pd(PPh_3)_2Cl_2$ (0.07 g, 0.09 mmol), PPh_3 (.01 g, 0.04 mmol), trimethylsilylacetylene (0.28 g, 2.85 mmol) and triethylamine (0.29 g, 2.85 mmol) in THF (15 mL), followed by CuI (4 mg, 0.02 mmol). After column chromatography a single fraction was obtained which gave a white solid assigned as compound **1A** (0.386 g, 80%). Mp 100-101 °C, IR (KBr, cm^{-1}): 2172, 1709; ^{1}H NMR (500 MHz, $CDCl_3$): δ 10.55 (s, 1H), 8.25 (dd, 1H, J=8.5, 1.0 Hz), 8.16 (dd, 1H,

J=8.5, 1.0 Hz), 7.93 (td, 1H, *J*=8.5, 1.0 Hz), 7.87 (td, 1H, *J*=8.5, 1.0 Hz), 0.37 (s, 9H); ^{13}C NMR (125 MHz, $CDCl_3$): δ 189.9, 146.0, 142.9, 140.5, 137.7, 133.3, 131.6, 130.4, 129.0, 105.1, 99.6, -0.5 (3C); MS: m/e (relative intensity): 256 (MH^++1, 23), 255 (MH^+, 100), 241 (25), 227 (10), 183 (17); Anal. Calcd for $C_{14}H_{14}N_2OSi$: C, 66.11; H, 5.55; N, 11.01. Found: C, 66.02; H, 5.69; N, 10.93.

1.3. Phenyl-(3-trimethylsilanylethynyl-2-quinoxalinyl)methanone (1B):

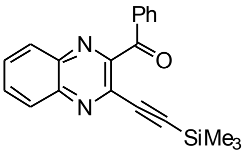

General procedure I was followed using (3-chloro-2-quinoxalinyl)phenylmethanone (**6B**) (0.61 g, 2.26 mmol), $Pd(PPh_3)_2Cl_2$ (0.08 g, 0.11 mmol), PPh_3 (.02 g, 0.06 mmol), trimethylsilylacetylene (0.33 g, 3.38 mmol) and triethylamine (0.34 g, 3.38 mmol) in THF (20 mL), followed by CuI (5 mg, 0.03 mmol). After column chromatography a single fraction was isolated as yellow thick liquid and assigned as compound **1B** (0.56 g, 75%). IR (KBr, cm^{-1}): 2157, 1677; ^{1}H NMR (500 MHz, $CDCl_3$): δ 8.18-8.12 (m, 2H), 7.97 (d, 1H, *J*=7.5 Hz), 7.91 (d, 1H, *J*=7.5 Hz), 7.90-7.82 (m, 2H), 7.65 (m, 1H), 7.51-7.47 (m, 2H), 0.09 (s, 9H); ^{13}C NMR (125 MHz, $CDCl_3$): δ 192.6, 153.6, 141.9, 139.6, 136.5, 135.4, 134.1, 131.6, 131.3, 130.3 (2C), 129.6, 129.2, 128.6 (2C), 140.7, 100.3, -0.8 (3C);

MS: m/e (relative intensity): 331 (MH^+, 100), 311 (5), 259 (5); Anal. Calcd for $C_{20}H_{18}N_2OSi$: C, 72.69; H, 5.49; N, 8.48. Found: C, 72.57; H, 5.59; N, 8.42.

2. General procedure II – coupling of carbene complex with alkynyl pyrazine/quinoxaline carbonyl derivatives and maleimides/dimethyl maleate

To a refluxing solution of alkynyl carbonyl derivative **1A** or **1C** (1 mmol) and maleimide/dimethylmaleate (1 mmol) in THF (5 mL), was added a solution of carbene complex **2** (1.1 mmol) in THF (10 mL) over a period of 1 h. After the addition was complete, the mixture was heated to reflux for a period of 12 h. The mixture was allowed to cool to room temperature and concentrated on a rotary evaporator. EtOAc (20 mL) was added and the residue filtered through celite (1.0 g). The solvent was removed on a rotary evaporator, and the crude products dissolved in ether (20 mL). To this solution of crude product in ether was added aqueous HCl (1:1) (0.5 mL) and the mixture stirred for 6 h at room temperature. The organic layer was separated. The aqueous layer was neutralized with saturated $NaHCO_3$ solution (3 mL) and extracted with ethyl acetate (3×10 mL). The combined organic layers (diethyl ether layer and ethyl acetate layer) was washed with water (3 mL) and brine (3 mL), and dried over anhydrous Na_2SO_4. Evaporation of solvent and purification by chromatography gave the pure products.

2.1. Coupling of carbene complex 2 with phenyl-(3-trimethylsilanylethynyl-2-pyrazinyl)methanone (1A) and *N*-phenylmaleimide (Table 1, entry 1).

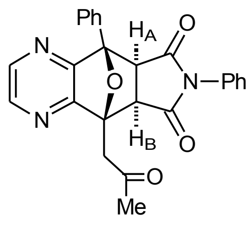

7a

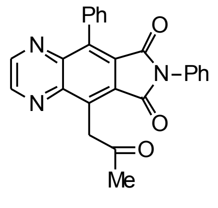

5a

General procedure II was followed using carbene complex **2** (98 mg, 0.39 mmol), alkynyl carbonyl derivative **1A** (100 mg, 0.35 mmol) and *N*-phenylmaleimide (62 mg, 0.35 mmol). The

crude product was purified using column chromatography (silica gel/ethyl acetate: petroleum ether 1:5) to yield the quinoxaline derivative **5a** (44 mg, 30%) and the oxa-bridged compound **7a** (64 mg, 42%) as yellow solids. *Compound* **7a**: Mp: 170 °C (decomposed); R_f (40% EtOAc/hexane) 0.53; IR (KBr, cm^{-1}): 1713, 1635; ^1H NMR (400 MHz, $CDCl_3$): δ 8.37 (d, 1H, J=2.8 Hz), 8.33 (d, 1H, J=2.8 Hz), 7.77 (d, 2H, J=7.6 Hz), 7.50 (t, 2H, J=7.6 Hz), 7.45-7.38 (m, 3H), 7.34 (m, 1H), 7.17 (d, 2H, J=7.6 Hz), 3.95 (d, 1H, J=6.9 Hz), 3.68 (d, 1H, J=18.0 Hz), 3.59 (d, 1H, J=18.0 Hz), 3.56 (d, 1H, J=6.9 Hz), 2.40 (s, 3H); ^{13}C NMR (125 MHz, $CDCl_3$): δ 202.9, 172.9, 171.2, 160.0, 159.1, 142.5, 142.3, 131.5, 131.3, 129.1 (2C), 128.9, 128.8, 128.3 (2C), 126.2 (2C), 126.1 (2C), 89.4, 85.3, 52.4, 50.3, 42.6, 30.6; MS: m/e (relative intensity): 426 (MH^+, 15), 408 (MH^+-H_2O, 40), 366 (50), 289 (45), 279 (40), 253 (100), 211 (18); Anal. Calcd for $C_{25}H_{19}N_3O_4$: C, 70.58; H, 4.50; N, 9.88. Found: C, 70.39; H, 4.65; N, 9.69. *Compound* **5a :** Mp: 160 °C (decomposed); R_f (40% EtOAc/hexane) 0.76; IR (KBr, cm^{-1}): 1714, 1634; ^1H NMR (500 MHz, $CDCl_3$): δ 8.99 (d, 1H, J=1.5 Hz), 8.95 (d, 1H, J=1.5 Hz), 7.58-7.48 (m, 5H), 7.46 (d, 2H, J=7.2 Hz), 7.44-7.36 (m, 3H), 5.08 (s, 3H), 2.50 (s, 3H); ^{13}C NMR (125 MHz, $CDCl_3$): δ 204.8, 166.6, 165.2, 146.5, 145.6, 144.8, 144.7, 141.1, 135.6, 132.1, 131.3, 130.7 (2C), 129.0 (2C), 128.9, 128.4, 128.1, 127.7 (2C), 126.6 (2C), 126.5, 40.3, 30.5; MS: m/e (relative intensity): 409 (MH^++1, 39), 408 (MH^+, 100), 380 (68), 335 (64); HRMS calcd for $C_{25}H_{20}N_3O_4$ (MH^+): 407.1270; found: 407.1270.

2.2. Coupling of carbene complex 2 with 3-trimethylsilanylethynylquinoxaline-2-carboxaldehyde (1C) and *N*-phenylmaleimide (Table 1, entry 3).

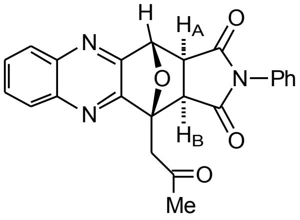

7c

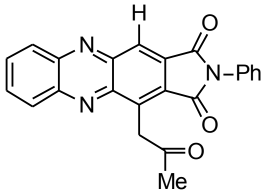

5c

General procedure II was followed using carbene complex **2** (108 mg, 0.43 mmol), alkynyl aldehyde **1C** (100 mg, 0.39 mmol) and *N*-phenylmaleimide (68 mg, 0.39 mmol). The crude product was purified using column chromatography (silica gel/ethyl acetate: petroleum ether 1:5) to yield the oxa-bridged compound **7c** (16 mg, 10%) and the phenazine derivative **5c** (78 mg, 52%) as white solids. In this case compound **7c** was readily converted to phenazine derivative **5c**. *Compound* **7c**: R_f (40% EtOAc/hexane) 0.76; IR (KBr, cm^{-1}): 1713, 1635; ^{1}H NMR (400 MHz, $CDCl_3$): δ 8.10 (d, 1H, *J*=4.8 Hz), 8.08 (d, 1H, *J*=4.8 Hz), 7.83-7.74 (m, 2H), 7.52-7.37 (m, 2H), 7.32 (d, 2H, *J*=7.6 Hz), 7.22 (d, 1H, *J*=8.0 Hz), 5.87 (s, 1H), 3.76 (d, 1H, *J*=7.0 Hz), 3.62 (d, 1H, *J*=18.0 Hz), 3.55 (d, 1H, *J*=18.0 Hz), 3.46 (d, 1H, *J*=7.0 Hz), 2.38 (s, 3H); ^{13}C NMR (150 MHz, $CDCl_3$): δ 202.3, 173.9, 173.1, 158.1, 157.4, 141.1, 140.8, 130.3, 130.1, 129.8, 129.3 (2C), 129.1, 128.9, 126.44, 126.4, 126.0, 86.9, 80.2, 49.2, 48.0, 42.2, 40.0; MS: m/e (relative intensity): 401 (MH^++1, 25), 400 (MH^+, 92), 383 (40), 382 (100, 340 (60); *Compound* **5c** (partial from the mixture of **7c** and **5c**): ^{1}H NMR (400 MHz, $CDCl_3$): δ 8.77 (s, 1H), 8.34-8.24 (m, 2H), 7.98-7.92 (m, 2H), 7.57-7.40 (m, overlapped with ArH of **7c**), 5.15 (s, 2H), 2.53 (s, 3H); ^{13}C NMR (150 MHz, $CDCl_3$): δ 131.5, 130.1, 129.8, 129.3, 126.5, 40.6, 30.7. *Compound* **5c :** Mp: 195 °C, R_f (40% EtOAc/hexane): 0.59; IR (KBr, cm^{-1}): 1714; ^{1}H NMR (400 MHz, $CDCl_3$): δ 8.77 (s, 1H), 8.35-8.23 (m, 2H), 7.96 (d, 1H, *J*=4.0 Hz), 7.95 (d, 1H, *J*=4.0 Hz), 7.59-7.39 (m,

5H), 5.15 (s, 2H), 2.53 (s, 3H); ^{13}C NMR (125 MHz, CDCl$_3$): δ 204.5, 166.6, 165.6, 144.7, 144.3, 144.1, 143.5, 137.5, 132.4, 132.2, 131.4, 130.5, 130.1, 129.8, 129.2 (2C), 128.6, 127.2, 126.6 (2C), 126.3, 40.6, 30.7; MS: m/e (relative intensity): 383 (MH$^+$+1, 30), 382 (MH$^+$, 100), 340 (47); Anal. Calcd for $C_{23}H_{15}N_3O_3$: C, 72.43; H, 3.96; N, 11.02. Found: C, 72.19; H, 4.15; N, 10.89.

2.3. Coupling of carbene complex 2 with phenyl-(3-trimethylsilanylethynyl-2-pyrazinyl)methanone (1A) and dimethyl maleate (Scheme 3).

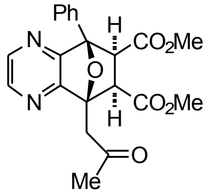

General procedure II was followed using carbene complex **2** (98 mg, 0.39 mmol), alkynyl carbonyl derivatives **1A** (100 mg, 0.35 mmol) and dimethyl maleate (50 mg, 0.35 mmol). The crude product was purified using column chromatography (silica gel/ethyl acetate: petroleum ether 1:5) to yield the oxa-bridged ketone **9A** (57 mg, 40%) as a thick yellow liquid.R$_f$ (40% EtOAc/hexane) 0.42; IR (KBr, cm^{-1}):1735, 1710; ^{1}H NMR (400 MHz, CDCl$_3$): δ 8.33 (bs, 2H), 7.94-7.87 (m, 2H), 7.52-7.39 (m, 3H), 3.95 (d, 1H, *J*=4.8 Hz), 3.78 (s, 3H), 3.71 (d, 1H, *J*=17.4 Hz), 3.51 (s, 3H), 3.46 (d, 1H, *J*=4.8 Hz), 3.33 (d, 1H, *J*= 17.4 Hz), 2.27 (s, 3H); ^{13}C NMR (75 MHz, CDCl$_3$): δ 203.0, 171.2, 169.9, 159.6, 158.1, 142.2, 141.9, 133.9, 129.0, 128.4 (2C), 126.9 (2C), 88.2, 85.3, 56.1, 53.0, 52.5, 52.3, 42.5, 30.9; MS: m/e (relative intensity): 379 (MH$^+$, 5), 347 (48), 335 (20), 252 (68), 211 (40); Anal. Calcd for $C_{21}H_{20}N_2O_6$: C, 63.63; H, 5.09; N, 7.07. Found: C, 63.85; H, 4.81; N, 7.25.

3. General procedure III – coupling of γ,δ-unsaturated Fischer carbene complex with alkynyl pyrazine/quinoxaline carbonyl derivatives.

To a refluxing solution of alkynyl carbonyl derivative **1A** or **1B** (1 mmol) in THF (10 mL) was added a solution of *γ,δ*-unsaturated carbene complex 10 (1.1 mmol) in THF (10 mL) over a period of 1 h. After the addition was complete, the mixture was heated to reflux for a period of 24 h. The mixture was allowed to cool to room temperature and concentrated on a rotary evaporator. EtOAc (20 mL) was added and the residue was filtered through celite (1.0 g). The solvent was removed on a rotary evaporator, and the residue stirred in silica gel/chloroform in the air for a further 2 h period and then filtered through a thin layer of celite. The solvent was removed on a rotary evaporator. Final purification was achieved by column chromatography on silica gel using ethylacetate/petroleum ether as eluent.

3.1. Coupling of carbene complex 10 with phenyl-(3-trimethylsilanylethynyl-2-pyrazinyl)methanone (1A) (Scheme 4).

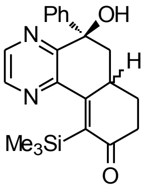

General procedure III was followed using carbene complex **10** (124 mg, 0.43 mmol) and alkynyl aldehyde **1A** (100 mg, 0.39 mmol). The crude product was purified using column chromatography (silica gel/ethyl acetate: petroleum ether 1:10) to yield alcohol **13A** (97 mg, 75%) as a white solid. Mp: 140 °C; R_f (40% EtOAc/hexane) 0.61; IR (KBr, cm^{-1}): 3417, 1649; ^1H NMR (500 MHz, $CDCl_3$): δ 8.61 (d, 1H, *J*=2.2 Hz), 8.58 (d, 1H, *J*=2.2 Hz), 7.33-7.27 (m, 3H), 7.05-7.00 (m, 2H), 4.15 (s, 1H), 2.58-2.47 (m, 2H), 2.43 (dd, 1H, *J*=12.8, 3.7 Hz), 2.38-2.20

(m, 2H), 1.93 (m, 1H), 1.82 (m, 1H), 0.20(s, 9H); ^{13}C NMR (125 MHz, $CDCl_3$): δ 203.7, 160.1, 157.2, 147.3, 146.6, 146.2, 145.2, 143.1, 128.8 (2C), 128.3, 127.0 (2C), 75.4, 45.7, 38.0, 35.3, 29.5, 2.9 (3C); MS: m/e (relative intensity): 365 (MH^+, 7), 350 (25), 349 (100), 279 (6); Anal. Calcd for $C_{21}H_{24}N_2O_2Si$: C, 69.19; H, 6.64; N, 7.69. Found: C, 69.01; H, 6.86; N, 7.76.

3.2. Coupling of carbene complex 10 with phenyl-(3-trimethylsilanylethynyl-2-quinoxalinyl)methanone (1B) (Scheme 4).

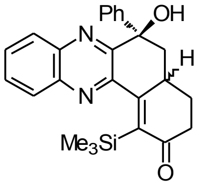

General procedure III was followed using carbene complex **10** (97 mg, 0.33 mmol) and alkynyl aldehyde **1B** (100 mg, 0.30 mmol). The crude product was purified using column chromatography (silica gel/ethyl acetate: petroleum ether 1:10) to yield alcohol **13B** (88 mg, 70%) as a white solid. Mp: 140 °C; R_f (20% EtOAc/hexane) 0.46; IR (KBr, cm^{-1}): 3417, 1657; ^1H NMR (500 MHz, $CDCl_3$): δ 8.90 (dd, 1H, *J*=3.5, 1.5 Hz), 8.04 (dd, 1H, *J*=3.5, 1.5 Hz), 7.86-7.79 (m, 2H), 7.29-7.24 (m, 3H), 7.02-6.98 (m, 2H), 4.61 (s, 1H), 2.61 (m, 1H), 2.59-2.52 (m, 2H), 2.41-2.31 (m, 2H), 2.01 (m, 1H), 1.88 (m, 1H), 0.26 (s, 9H); ^{13}C NMR (125 MHz, $CDCl_3$): δ 203.3, 160.1, 157.1, 147.2, 146.8, 146.6, 141.8, 141.3, 131.2, 130.9, 129.1, 129.0, 128.3 (2C), 127.7, 126.7 (2C), 75.2, 45.2, 37.7, 35.2, 29.3, 2.6 (3C); MS: m/e (relative intensity): 415 (MH^+, 29), 399 (100), 337 (62), 279 (79), 205 (37); Anal. Calcd for $C_{25}H_{26}N_2O_2Si$: C, 72.43; H, 6.32; N, 6.76. Found: C, 72.21; H, 6.51; N, 6.61.

References

1. Yoshida, K.; Otomasu, H. *Chem. Pharm. Bull.* **1984**, *32*, 3361–3365.
